# Obituary

**Published:** 1891-07

**Authors:** 


### Obituary.

Dr. Fordyce Barker died at his home in New York, Saturday, May 30, 1891, of apoplexy, in the seventy-third year of his age. Dr. Barker was so thoroughly identified with the profession of medicine, and had done so much to advance its lines of scientific research and improvement, that his name had become a household word with physicians, as well as the myriads of patients that he had ministered unto so successfully. Moreover, he was eminently a man of affairs, and so he will probably be more universally missed than any other American physician.

---

The death is announced of the distinguished Dr. Joseph Leidy, of Philadelphia, in the sixty-eighth year of his age, which took place April 30, 1891. Dr. Leidy was one of the most celebrated anatomists of the nineteenth century, and was in other respects a savant, of whose reputation the American profession has a right to be proud.—*Nashville Journal of Medicine and Surgery.*

